# Bone mineral density and body composition in normal weight, overweight and obese children

**DOI:** 10.1186/s12887-022-03317-y

**Published:** 2022-05-05

**Authors:** Samantha López-Peralta, Enrique Romero-Velarde, Edgar M. Vásquez-Garibay, Mercedes González-Hita, Laura C. Robles-Robles, Francisco J. Ruiz-González, Misael Alejandro Pérez-Romero

**Affiliations:** 1 Departamento de Reproducción Humana Crecimiento Y Desarrollo Infantil, Instituto de Nutrición Humana, Guadalajara, Jalisco México; 2 División de Pediatría del Hospital Civil de Guadalajara “Dr. Juan I. Menchaca”, Guadalajara, Jalisco México; 3 Departamento de Biología Molecular Y Genómica, Centro Universitario de Ciencias de La Salud de La Universidad de Guadalajara, Guadalajara, Jalisco México; 4 Clínica de Osteoporosis del Hospital Civil de Guadalajara “Fray Antonio Alcalde”, Guadalajara, Jalisco México

**Keywords:** Bone mineral density, Body composition, Overweight, Obesity, Children

## Abstract

**Background:**

There is a possibility that excess body fat affects bone mass gain and may compromise skeletal health in obese children. The purpose of the study was to identify the relationship between bone mineral density (BMD) and body composition in normal weight, overweight and obese children.

**Methods:**

This was a cross-sectional study of 6- to 11-year-old children who attended the hospital's outpatient clinic. They were apparently healthy and had no history of prematurity, low birth weight, or chronic diseases. Body mass index (BMI) was used to identify subjects as normal weight, overweight or obese. BMD and body composition were assessed by dual energy X–ray absorptiometry. The BMD values (total and lumbar spine) were compared between normal weight, overweight and obese children. Correlation coefficients were calculated, and multivariate models were performed.

**Results:**

Forty-nine children were included: 16 with normal weight, 15 that were overweight and 18 with obesity; the mean age was 8.4 ± 1.7 years. All the participants had a normal BMD (> – 2 SD). BMD was higher in obese children and had a positive correlation with total and trunk lean mass in the three study groups (*p* < 0.001). In obese children, an inverse correlation of lumbar spine BMD (Z score) with total and trunk fat mass (*p *< 0.05) was identified. In the multivariate models (with the whole group), the total lean mass was the only significant variable that explained BMD variability.

**Conclusions:**

BMD in obese children was higher than that in normal weight children, which is explained by their greater lean mass and not by excess body fat. In obese children, a higher fat mass was related to a lower lumbar spine BMD. Lean mass had a direct correlation with BMD in the three study groups and was the most important predictor of BMD, reflecting the importance of strengthening the muscular system through performing physical activity and practicing a healthy lifestyle.

## Introduction

The accumulation of bone minerals begins in intrauterine life and continues until the third decade of life, the culminating moment of development in which maximum bone mineralization is achieved [1, 2]. Childhood and adolescence are crucial stages for the development of optimal peak bone mass, which is the most effective measure to prevent osteoporosis [3]. Therefore, it is important to identify how different modifiable factors, such as body mass and body composition, can influence bone mineral density (BMD) during this period [4].

The prevalence of obesity in children and adolescents has increased globally in recent years, especially in low- and middle-income countries [5]. In Mexico, the combined prevalence of overweight and obesity in schoolchildren was 35.5% in 2018 [6].

Childhood obesity is associated with many health complications and an increased risk of premature onset of chronic diseases [5]. However, its effect on the accumulation of bone mass in children is not yet clear [7]. A systematic review with meta-analysis showed that overweight and obese children had significantly higher BMD than normal weight children [7]. However, it has also been noted that the bone mass of obese children and adolescents is insufficient for their body weight [8, 9] and that BMD is lower in adolescents with obesity [10]. During growth, lean mass has been directly related to BMD variability [11–14], while the effect of fat mass on BMD remains controversial [13].

Some studies have reported that the greater the amount of adipose tissue, the greater the total body BMD due to the increased mechanical load on the bone [7, 15]. However, body fat percentage and abdominal adipose tissue have also been shown to have a negative effect on BMD in overweight and obese children and adolescents [16, 17]. In addition, it has been reported that the excessive accumulation of trunk fat has a negative relationship with BMD, particularly when fat values exceed the 85^th^ percentile, for which the existence of a threshold above which body fat has a negative impact on BMD has been suggested [4].

There is a possibility that excess body fat affects bone mass gain and may compromise skeletal health in obese children [18, 19]. Therefore, it is important to understand the effects of body composition on BMD according to nutritional status, since the prevalence of overweight and obesity has increased dramatically in recent years [5].

Therefore, the objective of the present study was to identify the relationship between BMD and body composition in normal weight, overweight and obese children.

## Methods

This cross-sectional study included 56 children aged 6 to 11 years who attended the outpatient clinic of the Division of Pediatrics at the Hospital Civil de Guadalajara “Dr. Juan I. Menchaca”, Guadalajara, Mexico, from January to December 2018. Apparently healthy children were included, and informed consent was obtained from all participants and that of their parents/legally authorized representative of participants. Not included were subjects with a history of prematurity or low birth weight, a history of chronic, congenital or genetic diseases, who used steroid drugs for any reason, those with BMI/age below – 2 SD, or with physical data indicating that they would have started the pubertal growth spurt. These data were obtained in a questionnaire to the children and their mothers about physical changes that suggest the beginning of the pubertal growth spurt: incipient growth of mammary glands or appearance of pubic hair.

To calculate the sample size, the BMD data (g/cm^2^) reported by Rocher et al. [20] in obese children and their controls with an α of 0.05 and power of 0.80 were used. Nonprobabilistic sampling was carried out, including all the subjects who met the selection criteria in the indicated period of time. BMD was considered a dependent variable with values in g/cm^2^ and Z score, and body composition indicators were considered independent variables: fat mass (g); lean mass (g); and body fat percentage (%).

Once informed consent was obtained, the personal and family history of the study subjects, as well as a history of fractures (positive/negative; site and number of fractures) and history of clinically significant fracture (positive/negative), were obtained in a direct interview with the parents. A clinically significant fracture history refers to the presence of two or more long bone fractures at the age of 10 years and/or three or more long bone fractures at any age up to 19 years [21].

Weight and height measurements of the participants were taken to calculate the BMI/age to identify normal weight, overweight or obese subjects according to the 2007 World Health Organization (WHO) criteria [22]. The measurements were made in all cases by nutritionists trained in taking anthropometric measurements, and the anthropometric indices were calculated with the WHO Anthro Plus version 1.0.4 program.

Weight measurement was performed in the morning with a TANITA brand scale, model BF-682 (Tokyo, Japan), with a precision of 100 g. Subjects were weighed without shoes and as little clothing as possible, with an empty bladder and at least two hours after consuming food [23]. Height measurements were performed with a SECA brand stadiometer, model 213 (Hamburg, Germany), with a precision of 1 mm. The participant was measured barefoot and stood with heels together, legs straight and shoulders relaxed. The heels, calves, buttocks, scapulae, and back of the head should were to be in contact with the vertical surface of the stadiometer. The head was positioned in the Frankfort horizontal plane to slide the movable part of the stadiometer until it rested firmly on the head and pressed the hair, at which time the measurement was read [23].

Subsequently, dual energy X-ray absorptiometry (DEXA) was performed to evaluate BMD and body composition at the Osteoporosis Clinic of the Hospital Civil de Guadalajara "Fray Antonio Alcalde". The measurement was carried out with GE Lunar Prodigy Advance, USA equipment (operating system: enCORE V16). Data were collected on BMD and bone mineral content (BMC) of the total body excluding the head (BMD_TBLH_; BMC_TBLH_), BMD of the L1-4 region of the lumbar spine (BMD_L1-4_), lean mass (g), fat mass (g) and percentage of total body and trunk fat. During this study, the participant was accompanied by one of his or her parents.

Statistical analysis. Descriptive statistics were performed for quantitative variables (mean and standard deviation) and for qualitative variables (frequency and percentage). Data distribution was evaluated with the Shapiro–Wilk test. ANOVA was used to compare the mean values between groups (normal weight, overweight and obese) for the quantitative variables and chi square for the qualitative variables; the correlation between BMD and body composition was identified with Pearson's correlation test. Multiple regression models were performed to identify the best model that explains the variability of BMD.

Since this study required exposure to X-rays, although minimal, the parents or legal representatives of the child were informed about it, requesting their signature for informed consent. This research adhered to the guidelines of the Declaration of Helsinki (2013) and obtained the consent of the Ethics and Research Committee of the Hospital Civil de Guadalajara “Dr. Juan I. Menchaca” (Register: 0219/18 HCJIM/2018), and the informed consent from all participants and that of their parents/legally authorized representative.

## Results

Fifty-six participants aged 6 to 11 years were included; seven were excluded because they presented extreme values of the height/age index (*n* = 4) or BMD (*n* = 3). Of the 49 subjects analyzed, 16 had normal weight (32.7%), 15 were overweight (30.6%) and 18 were obese (36.7%). The distribution by sex was similar in the normal weight and overweight groups, while the percentage of boys was higher than that of girls in the obesity group, with no significant difference. The average age of all the participants was 8.4 ± 1.7 years. There was no significant difference when comparing age between the three study groups.

Table 1 shows the anthropometric variables of the participants. The average value of all the variables was higher in obese children, with a significant difference between groups, except for the height values (cm).

**Table 1:** Anthropometric variables according to body weight category

	Normal Weight (*n* = 16)	Overweight (*n* = 15)	Obesity (*n* = 18)	P^a^
	x (SD)	x (SD)	x (SD)	
Weight (kg)	27.79 (5.1)	36.75 (10.0)	49.33 (11.0)	< 0.001
Height (cm)	130.14 (10.0)	134.98 (13.2)	137.35 (10.6)	0.181
BMI (kg/m^2^)	16.29 (1.2)	19.72 (1.7)	25.92 (3.6)	< 0.01
Height/age (z score)	-0.24^b^ (0.8)	0.47 (0.9)	0.74 (0.9)	< 0.001
BMI/age (z score)	0.03 (0.7)	1.54 (0.3)	3.44 (1.6)	< 0.001

^a^ANOVA; ^b^ *p* < 0.05 vs obesity (post hoc analysis with Tukey test)

Six of the participants had a history of fracture; however, none of them presented a history of clinically significant fracture. All participants had a normal BMD (> – 2 SD) of the total body and the L1-4 region of the lumbar spine [21] (Table 2). When comparing BMD_TBLH_ and BMD_L1-4_ (Z score), significant differences were identified between the three groups, with higher values in children with obesity. In the first case, the increase was progressive and significant between groups of normal weight, overweight and obesity, while in the case of the lumbar region, it was higher in the obese participants compared to those of normal weight (*p *< 0.001); however, there were no significant differences in the comparison of participants with normal weight vs overweight, and overweight vs obesity (Table 2).

**Table 2:** Bone mineral content (BMC) and bone mineral density (BMD) of the total body excluding the head (TBLH) and the L1-4 region of the lumbar spine according to body weight category

		Normal Weight (*n* = 16)	Overweight (*n* = 15)	Obesity (*n *= 18)
		x (SD)	x (SD)	x (SD)
BMC	(g)	708.56 (179.3)	857.59 (256.2)	1006.44 (242.1)
BMD_TBLH_	(g/cm^2^)	0.62 (0.1)	0.68 (0.1)	0.74 (0.1)
	(z score)*	-0.67 (0.5)**	0.11 (0.7)***	0.76 (0.9)^†^
BMD_L1-4_	(g/cm^2^)	0.66 (0.1)	0.72 (0.1)	0.77 (0.1)
	(z score)*	-0.57 (0.5)	0.01 (1.0)	0.63 (0.9)^†^

* ANOVA *p *< 0.001; ** Normal Weight vs Overweight *p* < 0.01; *** Overweight vs Obesity *p* < 0.05; ^†^Obesity vs Normal Weight *p* < 0.001; Post hoc analysis with Tukey Test (Games-Howell test for Z score of BMD_L1-4_)

Table 3 shows the values of lean mass and fat mass (grams and %) that were significantly different between groups, with higher values in children with obesity. The mean total and trunk lean masses were higher in the obesity group than in the normal weight group (*p* < 0.001). Fat mass (grams and %) showed progressive and significant increases between the normal weight, overweight and obese groups.

**Table 3:** Lean and fat mass values according to body weight category

	Normal Weight (*n* = 16)	Overweight (*n* = 15)	Obesity (*n* = 18)
	x (SD)	x (SD)	x (SD)
**Lean mass** (g)*			
Total	19,341.81 (3008.6)**	22,241.93 (5623.6)	26,080.94 (5232.5)
Trunk	8874.63 (1240.0)**	10,064.8 (2626.0)	11,729.11 (2502.4)
**Fat mass** (g)*			
Total	7476.69 (2182.9)**	13,246.47 (4690.6)***	21,890.28 (6435.4)^†^
Trunk	2766.75 (1014.1)**	6090.0 (2531.7)***	10,608.50 (3831.2)^†^
**% Fat** *			
Total	26.42 (3.8)**	35.59 (5.5)***	43.91 (5.0)^†^
Trunk	22.62^e^ (5.3)**	35.99 (7.4)***	45.75 (6.8)^†^

* ANOVA *p* < 0.01 (Lean mass), *p* < 0.001 (Fat mass and % Fat);** Normal weight vs Obesity *p* < 0.01 (Lean and Fat mass), *p* < 0.001 (% Fat);*** Overweight vs Normal weight *p* < 0.01 (Fat mass), *p* < 0.001 (% Fat); ^†^Obesity vs overweight *p* < 0.01 (Fat mass), *p* < 0.001 (% Fat);(Post hoc analysis: Games-Howell test, Lean and Fat Mass; Tukey test, % Fat)

Table 4 shows the correlation coefficients of BMD and BMC with the components of body composition. Both BMD_TBLH_ (g/cm^2^) and BMC_TBLH_ (g) had a positive correlation with total and trunk lean mass in the three study groups (*p* < 0.001). In the same way, they correlated positively with the total and trunk fat mass in children with normal weight and overweight (*p* < 0.01). The obese participants had a different behavior: the BMC_TBLH_ (g) presented a direct and significant correlation with the total and trunk fat mass; however, the BMD_TBLH_ (g/cm^2^) did not correlate with the fat mass variables.

**Table 4:** Correlation of bone mineral content (BMC) and bone mineral density (BMD) with body composition in children according to body weight category

	Normal Weight (*n* = 16)				Overweight (*n* = 15)				Obesity (*n* = 18)			
	BMC_TBLH_^a^	BMD_TBLH_^a^	BMD _L1-4_^b^		BMC_TBLH_^a^	BMD_TBLH_^a^	BMD _L1-4_^b^		BMC_TBLH_^a^	BMD_TBLH_^a^	BMD _L1-4_^b^	
	g	g/cm^2^	g/cm^2^	z score	g	g/cm^2^	g/cm^2^	z score	g	g/cm^2^	g/cm^2^	z score
**Lean mass (g)**												
Total	0.937^***^	0.858^***^	0.560^*^	-0.340	0.959^***^	0.888^***^	0.581^*^	0.147	0.957^***^	0.822^***^	0.178	-0.325
Trunk	0.916^***^	0.842^***^	0.602^*^	-0.258	0.950^***^	0.867^***^	0.566^*^	0.143	0.912^***^	0.747^***^	0.090	-0.333
**Fat mass (g)**												
Total	0.793^***^	0.796^***^	0.479	-0.287	0.850^***^	0.715^**^	0.470	0.062	0.610^**^	0.456	-0.122	-0.512^*^
Trunk	0.703^**^	0.748^**^	0.409	-0.320	0.846^***^	0.727^**^	0.516^*^	0.120	0.485^*^	0.333	-0.249	-0.527^*^
**Fat (%)**												
Total	0.449	0.516^*^	0.228	-0.230	0.237	0.140	0.081	-0.092	-0.052	-0.132	-0.328	-0.423
Trunk	0.444	0.528^*^	0.193	-0.318	0.348	0.296	0.234	0.022	-0.092	-0.144	-0.377	-0.417

^a^ Total body less head; ^b^ L1-4 region of the lumbar spine; ***p < 0.001; **p < 0.01; *p < 0.05

Only in children with normal weight was a direct correlation observed between the BMD_TBLH_ (g/cm^2^) and the percentage of total and trunk fat (r = 0.516 and r = 0.528, *p* < 0.05, respectively). On the other hand, there was no significant correlation between BMD_TBLH_ (Z score) and body composition in any study group (data not shown in the table).

Regarding the relationship between the lumbar spine and body composition, BMD_L1-4_ (g/cm^2^) had a positive correlation with the total and trunk lean mass only in normal weight and overweight children (*p* < 0.05). In addition, it was directly related to trunk fat mass only in the overweight group (*p* < 0.05). In obese children, no significant correlation was identified between BMD_L1-4_ (g/cm^2^) and body composition. However, it was the only group in which a negative correlation of BMD _L1-4_ (Z score) with total and trunk fat mass was identified (r = – 0.512 and r = – 0.527, *p* < 0.05).

Three multivariate models were performed with all study participants (*n* = 49) in which BMD_TBLH_ (g/cm^2^), BMD_TBLH_ (Z score) and BMD_L1-4_ (g/cm^2^) were included as dependent variables; as predictor variables, sex, nutritional status (normal and overweight vs obesity), BMI/age, height/age, total lean mass (g), and percentage of total and trunk fat were included. Total lean mass was the only significant variable within the model that explained the variability of both BMD_TBLH_ and BMD_L1-4_ (g/cm^2^) (80.4% and 31.8%, respectively). Table 5 shows the results of the BMD_TBLH_ (Z score) model; in this case, 73.4% of the BMD variability was explained by BMI/age, the percentage of total fat and the height/age index.

**Table 5:** Multivariate analysis with BMD_TBLH_ as dependent variable; BMI-for-age, percentage of body fat, and height-for-age as independent variables

Dependent variable	Independent Variables	β	R^2^	*p*
BMD^a^	BMI/age^a^	0.710	0.493	< 0.001
	% Fat	-0.070	0.624	< 0.001
	Height/age^a^	0.339	0.734	< 0.001

*BMD* Bone mineral density; *TBLH* Total body less head; ^a^z score

## Discussion

The study of BMD and the variables that influence it in children and adolescents is important because in these stages of life, the accumulation of bone minerals must be optimized [2, 24]. In this study, all participants had a normal BMD (> – 2 SD) [21], reflecting good bone health despite differences in the nutritional status of the participants.

As expected, we identified significant differences in weight and BMI values between groups; and also in height, which was higher in children with obesity, as has been reported due to a higher growth velocity in the prepubertal stage, difference that decreases during puberty and that results in a similar final height between subjects with and without obesity [25].

When comparing the BMD_TBLH_ (Z score) between the three groups, we found a progressive increase as the category of nutritional status changed from normal weight to overweight and obesity; also, the BMD_L1-4_ (Z score) was higher in obese children compared to those of normal weight. These findings are consistent with what was reported in a systematic review with meta-analysis, which reported evidence (of moderate quality) that overweight and obese children have higher BMD than those of normal weight [7].

It has been noted that overweight and obese children have higher BMD because their higher body weight causes an increase in mechanical load on the bone, which stimulates bone shaping [12, 15, 26, 27]. However, as obese children generate greater muscle forces during physical activity [12, 28], bone strength seems to adapt more easily to dynamic muscular forces than to the static loads imposed by greater fat mass [29, 30]. According to this concept, Wetzsteon et al. (2008) [31] reported that although overweight and obese children had greater bone strength than those of healthy weight, it was not adapted to excess body fat but to greater muscle area. Gracia-Marco et al. (2012) [27] also concluded that overweight and obese adolescents had higher bone mass as a result of higher lean mass. Therefore, it is possible that obese participants in this study had higher BMD (total and lumbar spine) because their lean mass was greater compared to those of normal weight.

Regarding the relationship between BMD and body composition, a direct correlation was observed between lean mass and BMD_TBLH_ (g/cm^2^) in the three study groups. The BMD (g/cm^2^) of the lumbar spine had a direct relationship with the lean mass only in normal weight and overweight children. Similar to our study, several studies have reported a direct effect of lean mass on BMD in children and adolescents [11–14, 16, 32, 33].

In contrast to lean mass, the results of the relationship between body fat and BMD have been conflicting [13, 16, 17, 34]. In the present work, BMD_TBLH_ (g/cm^2^) correlated directly with the total and trunk fat mass only in normal weight and overweight children, with lower values in the latter group. In the same way, the percentage of total and trunk fat also correlated directly with the BMD_TBLH_ (g/cm^2^) only in children with normal weight. However, in obese children, BMD_TBLH_ (g/cm^2^) was not related to fat mass variables. We can speculate that as children accumulate body fat and go from normal weight to overweight and later to obese, the influence of fat mass on BMD decreases or disappears.

In the group of obese children, an inverse relationship between fat mass and lumbar BMD was identified: the greater the total and trunk fat mass, the lower the BMD_L1-4_ (Z score) (*p* < 0.05). Similar results were reported by Mosca et al. (2014) [16], who identified an inverse correlation between the percentage of body fat and BMD of the lumbar spine in overweight and obese adolescents. Similarly, Gallego et al. (2017) [35] reported that BMD of the total body and the lumbar spine decreased as the percentage of body fat and total fat mass increased. Recently, Rokoff et al. (2019) [4] identified that BMD_TBLH_ was directly related to trunk fat mass in children when fat values were below the 85^th^ percentile, while the BMD_TBLH_ decreased – 0.17 SD for each kg of increment in trunk fat mass in those who were above this value. Therefore, there is a possibility of a threshold above which central adipose tissue becomes more metabolically active and has a negative impact on bone [4].

These findings suggest that in normal weight and overweight children, a higher fat mass favors the accumulation of bone minerals; however, in children with obesity, it seems to be detrimental for the gain of adequate bone mass in the lumbar spine, compromising the future health of the skeleton in cases of persistent obesity.

The multivariate models in the total sample found that total lean mass was the only variable that explained the variability of both BMD_TBLH_ and BMD_L1-4_ (g/cm^2^). This finding aligns with previous studies that indicate that lean mass has a greater contribution to the variability of BMD than fat mass [12–14]. These findings highlight the importance of promoting and maintaining muscle mass through physical activity and a healthy lifestyle to optimize bone formation and maintain skeletal health [2, 13]. However, the BMD Z score model showed that 73.4% of the variability was explained by BMI, percentage of body fat and height-for-age index, which reflects the contribution of total body mass to BMD; in addition, the inclusion of height in the model could be explained by its relationship with age and with the BMI itself since, as noted, children with obesity showed higher height values.

The limitations of our study include the cross-sectional design and the lack of information on physical activity habits. Furthermore, the values obtained by DEXA are limited to measuring BMD (g/cm^2^) instead of volumetric BMD; the latter is important when the skeleton is still growing and bone size could influence BMD measurements.

One of the strengths of this work is the inclusion of the measurement of total body BMD and the L1-4 region of the lumbar spine, which are the sites recommended by the International Society for Clinical Densitometry for pediatric patients [21]. In addition, the analysis of the relationship between body composition and BMD according to nutritional status allowed us to identify how it changes as adiposity increases, which is reflected in changes in BMI.

In conclusion, the BMD in obese children was higher than that in normal weight children, which is explained by their greater lean mass and not by excess body fat. Fat mass had a direct relationship with BMD_TBLH_ in normal and overweight children; however, in the obesity group, a higher fat mass was associated with lower lumbar spine BMD. Lean mass had a direct correlation with BMD_TBLH_ in the three study groups and was the most important predictor of BMD versus fat mass, reflecting the importance of strengthening the muscular system through physical activity and practicing a healthy lifestyle.

## Data Availability

The datasets generated and/or analysed during the current study are available in the OSF repository, https://osf.io/.

## Abbreviations

BMC: Bone mineral content
BMD: Bone mineral density
BMI: Body mass index
DEXA: Dual energy X-ray absorptiometry
TBLH: Total body excluding the head
WHO: World Health Organization
